# Care of the chronic dialysis patient in the intensive care unit: a state-of-the-art review

**DOI:** 10.62675/2965-2774.20250130

**Published:** 2025-02-18

**Authors:** Julian Yaxley, Alexander Lesser, Victoria Campbell

**Affiliations:** 1 Department of Renal Medicine Logan Hospital Meadowbrook QLD Australia Department of Renal Medicine, Logan Hospital - Meadowbrook, QLD, Australia.; 2 Department of Intensive Care Medicine Gold Coast University Hospital Southport QLD Australia Department of Intensive Care Medicine, Gold Coast University Hospital - Southport, QLD, Australia.; 3 Sunshine Coast University Hospital Birtinya QLD Australia Department of Intensive Care Medicine, Sunshine Coast University Hospital - Birtinya, QLD, Australia.

**Keywords:** Dialysis, Intensive care, Renal replacement therapy, Intensive care units

## Abstract

Chronic dialysis patients account for a high proportion of intensive care unit admissions. The prevalent dialysis population is growing worldwide, accompanied by increasing medical complexity and comorbidities. Critical care physicians must be familiar with the unique clinical characteristics of this patient group. There is relatively little evidence specifically concerning the assessment and treatment of critically unwell individuals on long-term dialysis. This narrative review explores the approach to the management of chronic dialysis patients in the intensive care unit.

## INTRODUCTION

Patients receiving chronic dialysis account for approximately 5% of all intensive care unit (ICU) admissions in the United States and Europe.^(1)^ The prevalent hemodialysis (HD) population has grown by 50% worldwide over the past decade, and further doubling is predicted over the next decade.^(2,3)^ In many nations, the average age and comorbidity of this patient group has also increased. Chronic dialysis patients present unique medical challenges in the ICU and are associated with poorer outcomes.

Intensive care medicine specialists are providing care for chronic dialysis patients with increasing frequency. However, there are no published guidelines specifically dedicated to the management of chronic dialysis patients with critical illness or those in the ICU. Intensivists may be less familiar with the application of HD in chronic dialysis patients than with the provision of acute renal replacement therapy (RRT).

This narrative review discusses the clinical approach to chronic dialysis patients in the ICU based on the best available evidence. The paper is primarily aimed at critical care physicians who assume the care of chronic dialysis patients in a closed ICU model, in which ICU staff are the primary prescribers of RRT. The term ‘chronic dialysis patient’ is used throughout to refer to individuals with end-stage renal failure receiving long-term, maintenance, intermittent HD (IHD).

### Epidemiology and outcomes

Compared with the general population, chronic dialysis patients have higher rates of ICU admission.^(4)^In Australia, for example, 2 - 8% of the prevalent HD population is admitted to an ICU annually, equating to a 36-fold relative risk of ICU admission compared with the nondialysis population.^(5,6)^ The risk is even greater among indigenous Australians and in regional and rural centers.^(7,8)^ Chronic dialysis patients have greater disease severity at ICU admission, longer ICU stays, increased likelihoods of ICU readmission during the same hospitalization, and increased ICU mortality.^(4,9,10)^The risk of death in the ICU appears to be at least 50% greater in chronic dialysis patients than in patients without renal impairment.^(9-11)^The main reasons for ICU referrals in most datasets are sepsis and acute pulmonary edema.^(10,12)^

Chronic dialysis patients also have poorer long-term outcomes following ICU admission. Fewer than half of the patients survive longer than 6 months,^(4,13)^and the likelihood of hospital readmission after discharge is threefold greater than that of matched controls.^(14)^ Rates of postintensive care syndrome, characterized by poor cognitive and physical function following critical illness in the ICU, are also likely increased, although precise data are lacking. In a cohort study of more than 25,000 consecutive patients treated in American ICUs, HD was significantly associated with the subsequent development of cognitive impairment (hazard ratio [HR] 1.70; 95% confidence interval [95%CI] 1.30 - 2.23).^(15)^ Implicated factors include frailty with a reduced physiologic reserve, malnutrition, and immunosuppression.

Illness severity predictive scoring systems, such as the Acute Physiologic and Chronic Health Evaluation (APACHE) and Sequential Organ Failure Assessment (SOFA), are generally reliable in subjects with end-stage renal failure.^(10,16)^ However, there is a positive selection bias associated with outcome data, in that high-dependency support is generally offered only to a subset of chronic dialysis patients who are considered healthier.

Intensive care unit clinicians should appreciate the adverse quality-of-life and mortality outcomes of critical illness in chronic dialysis patients. Advance care planning with shared decision-making and clear goal-setting are important. Many chronic dialysis patients are realistic about their prognosis and prefer a conservative rather than an aggressive or invasive approach.^(17)^

### Indications for dialysis

Emergency indications for dialysis initiation in acute kidney injury (AKI) are well known. Classic examples include refractory hyperkalemia or volume overload. Chronic dialysis patients who have missed treatment or who develop complications between routine sessions can develop the same emergent indications. Regular outpatient dialysis schedules should be replicated where possible to maintain homeostasis and prevent complications. Extra treatments might be necessary preemptively when deterioration or invasive interventions are anticipated or when high fluid volumes are administered. The utility of HD following radiocontrast exposure is controversial.

### Volume status assessment

Hypervolemia is a constant danger in chronic dialysis patients. Because fluid is frequently administered in large volumes in the ICU, vigilance is needed to prevent serious volume overload. Assessment of volume status can be particularly challenging in the chronic dialysis patient population. Knowledge of the individual’s ‘target’ weight, defined as their euvolemic weight goal during chronic HD, is informative.^(18)^ However, the target weight can be misleading in the context of third-spacing in critical illness. The physical examination should focus on jugular venous pressure, crepitations on lung auscultation, ascites, and leg edema. A physical examination is helpful in the correct clinical context.^(19-21)^ Although studies of the utility of brain natriuretic peptide and transthoracic echocardiogram have produced mixed results,^(22-25)^chest radiography can accurately detect cardiogenic pulmonary edema regardless of dialysis status.^(26-28)^Lung ultrasound, which searches for signs of pulmonary edema, is emerging as a useful tool for setting ultrafiltration targets. Compared with usual care, small randomized trials demonstrated significantly improved long-term blood pressure control and weight reduction in chronic dialysis patients when evaluated with lung ultrasound.^(29,30)^ The best way to incorporate these strategies into the care of patients in the ICU is unclear.

### Fluid resuscitation

No prospective studies have specifically examined fluid resuscitation in chronic dialysis patients. Potential complications of fluid replenishment in people without residual urine output include hypervolemia and hyponatremia. However, chronic dialysis patients may respond differently to fluid therapy than the wider population because of their altered baseline physiology. Retrospective analyses revealed that chronic dialysis patients consistently receive lower fluid volumes in sepsis than their matched peers.^(31)^ In a secondary analysis of the Protocolized Care for Early Septic Shock (ProCESS) trial, a multicenter randomized controlled trial (RCT) of 1,341 participants with septic shock allocated to protocolized resuscitation versus standard care,^(32)^ outcomes in the chronic dialysis patient subgroup were independent of the fluid volume received.^(33)^ Similarly, a post hoc analysis of the major RCT by Rivers et al.^(34)^ investigating early goal-directed therapy suggested a disproportionate mortality benefit among the small number of included chronic dialysis patients versus other trial participants.^(35)^ Several retrospective cohort studies have demonstrated no relationship between a liberal or restrictive fluid resuscitation strategy and rates of endotracheal intubation, hospital length of stay or mortality in septic chronic dialysis patients.^(36)^ Although promising, the literature is currently insufficient to recommend early vasopressor use or goal-directed therapy in chronic dialysis patients with shock. Practices must continue to be individualized while further research is awaited.

### Renal replacement therapy modalities and vascular access

There is no evidence to support the best way to deliver RRT in the ICU. Prescriptions must be individualized, particularly for chronic dialysis patients. A clear knowledge of the components of the RRT prescription aids the intensivist’s understanding of the baseline therapy of chronic dialysis patients and how to complement it during critical illness, such as with adaptation to continuous RRT (CRRT).

### Dialysis kinetics

A complete overview of the dialysis kinetics is beyond the scope of this article. However, the fundamental principles of an RRT prescription are applicable to both IHD and CRRT. Although IHD is used by convention as an umbrella term, HD technically defines diffusive solute transfer across a semipermeable dialyzer membrane. Practically, however, IHD also frequently incorporates convective therapies. The most common treatment schedule for IHD is three sessions per week, at 4 hours per session, whereas CRRT is defined as continuous therapy for at least 24 hours.

Compared with a blood flow of 100 - 150mL/minute for CRRT, outpatient IHD typically utilizes a blood flow rate of at least 300mL/minute. Dialysate flow rates in intermittent HD approach 500mL/minute, compared with approximately 30mL/minute in CRRT.^(12)^ The dialysate usually runs in a countercurrent direction in both IHD and CRRT. Clearance is relatively more dependent on the blood flow rate in IHD as opposed to the dialysate flow rate in CRRT. Not all dialyzers are compatible with both IHD and CRRT.

Convective solute clearance through hemofiltration or hemodiafiltration enhances middle molecule transport and can be incorporated into both IHD and CRRT. Both require substitution with replacement fluid to account for excess ultrafiltration; the amount and composition of the replacement fluid are variable. Long-term hemodiafiltration may provide a slight mortality benefit over chronic IHD but is uncommonly used worldwide owing to cost constraints. Intermittent hemofiltration is not prescribed because solute clearance is inadequate within short treatment times. In CRRT, the evidence has thus far not demonstrated that hemofiltration or hemodiafiltration is superior to HD alone.

Ultrafiltration is best defined as the convective clearance of plasma solutes and water;^(37)^however, the term ‘ultrafiltration’ is described inconsistently across nephrology and critical care texts and can cause confusion. Ultrafiltration techniques, in which volume is removed but plasma solute concentrations are largely unchanged because replacement fluid substitution is omitted, are common to both IHD and CRRT. Ultrafiltration is associated with less hemodynamic instability than HD or hemodiafiltration and is often interspersed within dialysis treatments.^(38)^ In the context of IHD, ultrafiltration alone is referred to as isolated ultrafiltration, whereas in CRRT, it is known as slow continuous ultrafiltration. In general, the maximal volume removed during IHD and CRRT is approximately 1L per hour and 150mL per hour, respectively.

### Dialysis dose

The dose refers to the volume of blood purified by the dialysis process. The operating definition varies between IHD and CRRT. In CRRT, the dose is typically represented by the effluent volume, measured in mL/kg/hour. The dose in IHD, also called adequacy, is typically calculated using equations that estimate urea clearance, such as Kt/V (where K = urea clearance, t = treatment time in minutes, and V = urea volume of distribution) or the urea reduction ratio. The dose formulas used in CRRT and IHD are not reciprocal because urea kinetics differ between patient groups and between slow and rapid therapy.^(39,40)^

The dialysis dose is a product of the flow rate and therapy time. In patients with AKI, high-quality evidence supports a CRRT dose of 25 - 35mL/kg/hour.^(41-43)^Intermittent HD with a thrice-weekly Kt/V of 1.2 - 1.4 has also been shown to be adequate in the setting of AKI;^(44,45)^ however, extra sessions of isolated ultrafiltration may be needed on nondialysis days. The recommended maintenance goal for chronic dialysis patients not in the ICU is also a Kt/V of 1.2 - 1.4 three times per week or a urea reduction ratio of 60 - 65% per session because of data showing improved mortality.^(46)^

### Intermittent *versus* continuous renal replacement therapy in the intensive care unit

Although trials have demonstrated no differences in kidney or mortality outcomes between HD modalities when initiated for AKI, their methodology and conclusions have been criticized.^(47,48)^Because CRRT is typically preferred in unstable ICU patients, studies are flawed by exclusion or transfer into the CRRT arm of the sickest patients. In some RCTs, approximately 10% of participants allocated to IHD required switching to CRRT because of hemodynamic instability.^(49,50)^The advantages of CRRT include superior hemodynamic stability^(51)^ and cerebral perfusion^(52)^and more predictable drug dosing. Compared with systemic heparin anticoagulation, the recent wider use of regional citrate anticoagulation during CRRT has also led to lower rates of bleeding and circuit clotting.^(53)^ Given the experience and data thus far, CRRT appears to be the preferred modality in hemodynamically unstable patients, whereas IHD is a suitable step-down option.

Renal replacement therapy is often dictated by pragmatic issues such as center resources. Continuous RRT is increasingly used, but there is significant interregional variation, and IHD remains the prevailing modality in ICUs worldwide.^(12)^ Continuous RRT accounts for the majority of RRT delivered in ICUs in Australia and New Zealand, whereas IHD is the predominant modality in the United States. Intermittent HD is more cost-effective in most centers. Hybrid techniques, commonly referred to as prolonged intermittent renal replacement therapy or sustained low-efficiency dialysis, are also increasingly encountered and supported by evidence. Sustained low-efficiency dialysis uses IHD technology to deliver therapy more slowly and may represent a valid alternative in many circumstances. Systemic heparin and regional citrate anticoagulation are both suitable methods for anticoagulation during sustained low-efficiency dialysis.^(54)^

### Peritoneal dialysis

In most countries, a minority of patients receive RRT in the form of peritoneal dialysis (PD). In Australia, for example, PD is utilized by 22% of prevalent dialysis patients.^(55)^The relative incidence of ICU admission and median ICU length of stay appear to be similar between PD patients and IHD patients, although the clinical presentations differ. In one study, the most frequent reason for ICU admission among PD patients was for postoperative care.^(56)^Urgent-start PD is rarely performed.

In the ICU, many individuals on PD can continue their routine regimen successfully. The nephrology service assists with PD nursing and titration. Clearance and ultrafiltration are dictated by the PD dialysate concentration and the volume and number of exchanges. Peritoneal dialysis is inadequate in some conditions, whereby temporary conversion to HD is unavoidable. Peritoneal dialysis acts slowly in achieving ultrafiltration and biochemical control and relies on a functioning peritoneal membrane. Relative contraindications include recent abdominal surgery, intraabdominal sepsis or other acute processes, respiratory failure, and extreme metabolic derangements. In a Canadian registry study, the likelihood of permanent transfer to HD was 12% if a PD patient was admitted to the ICU.^(57)^

### Renal replacement therapy access

Chronic dialysis patients usually have an upper limb arteriovenous native fistula or synthetic graft. Their pre-existing arteriovenous access (AVA) should be utilized for IHD when feasible. Continuous RRT should not be routinely performed via AVAs because needle dislodgment, infiltration, thrombosis or hematoma may lead to damage or loss of the access. Fatal exsanguination incidents have been reported. In a retrospective study of ICU patients receiving CRRT via AVA for a median of 4 days, the rate of irreparable access loss was 6% and an additional 10% of patients required central venous catheter (CVC) placement due to mechanical AVA complications.^(58)^ Sustained low-efficiency dialysis could be performed through an AVA, but this option awaits further study. The use of an AVA requires a trained dialysis nurse with vigilant monitoring of the site throughout treatment.

If a CVC must be placed acutely for HD in subjects with a functioning AVA, such as for CRRT or when no trained personnel are available for cannulation, a femoral nontunneled line may be reasonable. This prevents traumatic injury to the jugular and subclavian veins, maintaining patency for future purposes. In most cases, the shorter dwell time allowed by femoral catheters should be sufficient to stabilize the patient to a point where reversion to IHD via their existing AVA is acceptable. If an AVA fails, a tunneled HD catheter is inevitably needed and is preferred upfront to circumvent unnecessary procedures. Tunneled CVCs are placed by critical care physicians in some centers. The right internal jugular vein is the first-line cannulation approach for tunneled lines in most instances.

### General vascular access

Securing reliable intravenous access can be problematic in chronic dialysis patients with an extensive history of vascular interventions. The impact on current or future AVAs should be considered. Central venous access is typically obtained via the jugular or femoral vein. Subclavian CVCs are avoided ipsilateral to a current or future AVA. Tunneled small-bore CVCs are being explored as an alternative to peripherally inserted central catheters. Peripherally inserted central catheter placement is associated with central vein stenosis and upper limb deep vein thrombosis and is an independent predictor of future adverse AVA outcomes.^(59,60)^

Phlebotomy, peripheral intravenous cannulation, or the placement of an intra-arterial cannula near a current or anticipated AVA is discouraged. Cannulation attempts are preferred distal to the site, such as in veins on the dorsum of the hand, rather than proximal. Anecdotally, preserving vessel integrity appears to increase AVA maturation and longevity. Arteriovenous access should also be protected from accidental compression in ventilated patients and from the application of armbands or blood pressure cuffs over the site.

Direct cannulation of an AVA is not contraindicated in emergency conditions where there is no alternative. Indeed, rapidly accessing an AVA may be prudent since stasis in the setting of prolonged hypotension is an independent risk factor for AVA thrombosis.^(61)^ The steps of AVA cannulation are similar to those of peripheral intravenous cannulation: an upper arm tourniquet is tightened to distend the vessel, after which the cannula is inserted into the outflow venous limb of the AVA in a downstream direction. The findings of blood gas samples from the AVA are almost indistinguishable from those of radial arterial blood gas samples.^(62)^

### Intradialytic emergencies

Life-threatening intradialytic complications, either in the ICU or the outpatient dialysis unit, are rarer among chronic dialysis patients than among those acutely commencing HD. Emergencies may be related specifically to dialysis or represent the exacerbation of an underlying or unrelated condition. Because evidence-based data are scarce, interventions in the ICU should be made on a case-by-case basis, and core resuscitation principles should be prioritized.

### Hypotension

Intradialytic hypotension is typically mild and is explained by volume and osmotic changes. Judicious crystalloid fluid boluses and reduced ultrafiltration restore blood pressure in most instances. However, dialysis-related hypotension can sometimes be profound; differential diagnoses for these severe cases include sepsis, decompensated heart failure, and myocardial infarction. Temporary cessation of HD is often necessary while the patient is stabilized. Persistent hypotension necessitates intravenous vasopressor support to facilitate further treatment. Vasopressor choices are analogous to those of the general population. Intravenous calcium may be a helpful adjunct. Because most chronic dialysis patients are conditioned to hypertension at baseline, the preferred acute blood pressure goals are uncertain.

### Anaphylaxis

The incidence of anaphylaxis is less than 4 cases per 100,000 RRT treatments.^(63)^ Adverse allergic reactions can occur after exposure to any part of the HD apparatus or to medications administered during treatment. The therapeutic approach to intradialytic anaphylaxis mirrors other settings. Renal replacement therapy should be stopped, and contaminated fluid or blood should be discarded. Further dialysis is absolutely contraindicated until the patient is stabilized, potential sources are removed, and fresh equipment is available.

### Neurologic disturbances

Intradialytic encephalopathy manifests on a spectrum ranging from mild confusion to seizures or coma. Uremic encephalopathy and dialysis disequilibrium syndrome are the main concerns. Uremic encephalopathy is neurologic dysfunction caused by the toxic effects of uremia, whereas disequilibrium syndrome is thought to be related to precipitous urea clearance and osmotic shifts that induce cerebral edema. The dialysis prescription should be slowed or paused in cases of intradialytic encephalopathy. Continuous RRT is probably better tolerated than IHD for this purpose. Empiric hypertonic saline or mannitol may be indicated in unstable patients to reverse cerebral edema.

### Cardiac arrest

The incidence of cardiac arrest is approximately 1 event per 20,000 outpatient IHD treatments.^(64)^The relative risk of intradialytic cardiac arrest is likely greater in the ICU because of predisposing factors. Most cases involve nonshockable rhythms. The etiology of dialysis-related cardiac arrest is thought to primarily reflect hypotension and the arrhythmogenic state. Advanced life support should be provided in accordance with professional guidelines. However, some nuances specific to the dialysis setting may be beneficial. Empiric pharmacologic treatment for acidemia and hyperkalemia is usually appropriate. Other interventions specific to intradialytic cardiac arrest may also include immediate cessation of dialysis, disconnection of unnecessary tubing, and a fluid bolus.

### Infections in chronic dialysis patients

Long-term dialysis results in increased susceptibility to infection.^(65)^ The likelihood of infection with multiresistant organisms is particularly high.^(66)^ Sepsis is more common in chronic dialysis patients than in the general population and is associated with a significantly greater relative risk of death.^(9,67)^The most frequent sources of sepsis associated with ICU admission in chronic dialysis patients are pneumonia and cellulitis,^(9)^ whereas the leading causes of infection-related death are vascular access infection and respiratory tract infection.^(37)^ Hemodialysis vascular access should be examined as a potential source in patients with bacteremia. Antibiotic agents must be dosed with reference to intrinsic renal elimination and drug dialysability. Intravenous antibiotics administered with intermittent HD are a convenient option in appropriate circumstances.

### Daily intensive care unit checklist

Daily rounding in the ICU should incorporate a range of evidence-based preventative interventions, often using standardized checklists. The most recognized checklist is FAST HUG (feeding, analgesia, sedation, thromboprophylaxis, head up, ulcer prophylaxis, glucose).^(68)^ The best practices for chronic dialysis patients are reviewed in table 1.

**Table 1:** Routine supportive care for chronic dialysis patients in the intensive care unit

Feeds and fluids	− Malnutrition is highly prevalent − Enteral and parenteral nutrition are well-tolerated but can result in fluid overload necessitating additional dialysis. Diuretic responsiveness is poor. In the community, dialysis patients usually require a daily fluid restriction to 1L − No robust evidence supports a ‘renal’ dietary formula (i.e., concentrated, electrolyte-restricted) over standard nutritional supplementation − Nonwater body mass is lost rapidly through deconditioning. The target weight must be adjusted to avoid hypervolemia
Analgesia and sedation	− Renal insufficiency affects the pharmacokinetics and pharmacodynamics of some anesthetic and analgesic agents. Drugs that are not renally cleared are preferable − Propofol is not markedly altered by end-stage renal failure and can be titrated to effect^(69)^ − There is interindividual variability in the response to opiates; short-acting agents such as fentanyl are less affected by renal impairment than long-acting alternatives such as morphine^(70-71)^ − Midazolam is minimally affected by kidney function in bolus doses but accumulates with longer infusions
Thromboprophylaxis	− There is an increased incidence of venous thromboembolism − Chemical venous thromboembolism prophylaxis may be associated with reduced mortality^(72)^ − At our hospital we frequently give subcutaneous unfractionated heparin 5,000U twice daily or enoxaparin 20mg daily
Ulcer prophylaxis	− Renal replacement therapy is a risk factor for stress ulcer bleeding − Routine pharmacologic ulcer prophylaxis is of uncertain benefit
Bowel management	− Polyethylene glycol laxatives and sodium phosphate enemas may induce electrolyte and acid–base disturbances and volume overload
Indwelling catheters	− Observational studies show higher infection rates for temporary jugular and femoral hemodialysis central venous catheters beyond approximately 21 and 7 days, respectively.^(73)^ However, the optimal time for preemptive central venous catheter exchange is unknown − Tunneled peritoneal dialysis catheters may be complicated by exit-site infection or peritonitis. Peritoneal dialysis catheter removal is performed by the interventionalist or surgeon − Peripheral cannulas sited near an arteriovenous access should be relocated when appropriate

### Perioperative care

The perioperative management of chronic dialysis patients can be challenging. This cohort has higher rates of surgery and is more likely to require postoperative ICU admission than the matched population.^(74,75)^Postoperative morbidity and mortality are also greater, particularly after unplanned surgery.^(76,77)^Plans should be made for RRT before and after surgery, as adequate perioperative dialysis is associated with fewer complications, such as infection, wound breakdown, bleeding and pulmonary edema.^(37,78,79)^Transfusion requirements may be raised due to impaired erythropoiesis. Aggressive erythropoietin dosing for acute anemia is ineffective in the short term. Pharmacologic reversal agents for uremic bleeding include tranexamic acid and desmopressin. Renal replacement therapy anticoagulation may need to be minimized. Intravenous fluids must be used cautiously to avoid hypervolemia. Compared with balanced crystalloid solutions, 0.9% sodium chloride slightly increases the incidence of hyperchloremic acidosis but not hyperkalemia.^(80,81)^

### Kidney transplantation

Many chronic dialysis patients are candidates for future kidney transplantation. Pretransplant status may affect therapy decisions in the ICU. For example, blood transfusion leads to antigen sensitization, negatively impacts graft outcomes, and should be avoided where possible.^(82)^ Leuko-depleted blood products are preferred.^(82,83)^Temporary coverage with an oral immunosuppressive agent, such as cyclosporin, reduces antibody formation and is still recommended by some centers on the basis of very low-quality evidence.^(84,85)^Consultation with nephrology is crucial. Femoral CVCs or arterial lines are discouraged to preserve the iliac vessels for future transplant surgery or to avoid anastomotic or embolic injury to a current graft.

### Directions for future research

Resuscitation strategies for chronic dialysis patients are debated. There is no convincing evidence for a specific approach in this patient group. The best implementation of fluids and vasopressors may be different in the context of unique baseline physiology. The relevance of endothelial glycocalyx abnormalities in patients with sepsis and chronic kidney disease is also attracting interest.^(86)^ Both the measurement of glycocalyx degradation to guide fluid resuscitation and the use of the glycocalyx as a therapeutic target are being investigated.^(87)^

Extracorporeal blood purification is being investigated for its therapeutic role in septic shock. Hemoperfusion or high-dose, high-flux hemofiltration can be utilized for cytokine and endotoxin removal. The fact that the therapy itself activates inflammatory mediators is a limitation. Other problems include hematologic and metabolic derangements. Extracorporeal purification is not yet supported by rigorous data, and further research is warranted. Compared with other groups, there may be greater utility in septic chronic dialysis patients with preexisting vascular access and long-term exposure to extracorporeal therapy.

Health care resource utilization should be a focus of future studies. There is an overreliance on ICUs to accommodate chronic dialysis patients. Intensive care unit admissions are frequently brief and focus on expediting RRT. Better utilization of acute medical or coronary care units may be possible in cases of lower complexity. Modifications to ward resourcing and staffing models, particularly to increase the capability for after-hours dialysis, may reduce the need for ICU referrals. The contest between open and closed high-dependency models remains controversial,^(88)^ likely depends on regional factors, and should be further evaluated.

## CONCLUSION

Caring for chronic dialysis patients in the intensive care unit can be challenging. The heterogeneity of this patient cohort, including their altered baseline physiology, and a lack of supporting evidence, highlights the importance of individualized care. The appreciation of patients’ baseline intermittent hemodialysis regimens can inform tailored provision of renal replacement therapy in the intensive care unit. A structured approach to routine intensive care unit care, such as deep vein thrombosis prophylaxis and antibiotic stewardship, is also important. Resuscitation strategies and improving the utilization of hospital resources by chronic dialysis patients are priorities for future research.
